# Research on the Total Channel Capacities Pertaining to Two Coverage Layouts for Three-Dimensional, UAV-Assisted Ad Hoc Networks

**DOI:** 10.3390/s23073504

**Published:** 2023-03-27

**Authors:** Xiao Yan, Shenglong Zhu, Qian Wang, Hsiao-Chun Wu

**Affiliations:** 1 School of Aeronautics and Astronautics, University of Electronic Science and Technology of China, Chengdu 611731, China; 2 Aircraft Swarm Intelligent Sensing and Cooperative Control Key Laboratory of Sichuan Province, Chengdu 611731, China; 3 School of Electrical Engineering and Computer Science, Louisiana State University, Baton Rouge, LA 70803, USA

**Keywords:** 6G wireless communications, UAV-assisted wireless networks, total channel capacity, stochastic geometry, square cell coverage layout, hexagonal cell coverage layout

## Abstract

Unmanned aerial vehicles (UAVs) employed as airborne base stations (BSs) are considered the essential components in future sixth-generation wireless networks due to their mobility and line-of-sight communication links. For a UAV-assisted ad hoc network, its total channel capacity is greatly influenced by the deployment of UAV-BSs and the corresponding coverage layouts, where square and hexagonal cells are partitioned to divide the zones individual UAVs should serve. In this paper, the total channel capacities of these two kinds of coverage layouts are evaluated using our proposed novel computationally efficient channel capacity estimation scheme. The mean distance (MD) between a UAV-BS in the network and its served users as well as the MD from these users to the neighboring UAV-BSs are incorporated into the estimation of the achievable total channel capacity. We can significantly reduce the computational complexity by using a new polygon division strategy. The simulation results demonstrate that the square cell coverage layout can always lead to a superior channel capacity (with an average increase of 7.67% to be precise) to the hexagonal cell coverage layout for UAV-assisted ad hoc networks.

## 1. Introduction

Unmanned aerial vehicles (UAVs) have surfaced as a pioneering technology in the 21st century and are exceptionally apt for military operations, including search-and-rescue missions, aerial photography, and structural inspections [1]. In addition, UAVs and UAV-assisted wireless networks have already been proven to be invaluable in civil applications, such as monitoring fires, transporting goods, detecting oil leakages, and providing network services [2]. UAVs may be employed as mobile base stations (BSs) or relays as they can be deployed for flexible coverage areas on demand, especially for future sixth-generation (6G) wireless networks [3]. UAV-BSs can enable the existing wireless communication networks to increase another dimension such that future wireless networks can be three-dimensional in space [4,5,6], particularly in situations where it is difficult or impossible to connect nodes using the conventional terrestrially based network infrastructure. However, the physical limitation of a UAV’s battery capacity restricts its flight time [5], making it impossible for a UAV-BS to provide continuous communication service as a terrestrial base station. Nevertheless, persistent communication service can still be ensured by replacing a battery-depleted UAV-BS with a fully charged one in a UAV-assisted wireless network. In addition to the aforementioned battery limitation, for a UAV-assisted ad hoc network, a main challenge is to choose an appropriate coverage layout, namely a proper cell partition, to maximize the network capacity. In typical scenarios, such as on-demand hot spot enhancement and post-disaster emergency communications [5,7,8], the UAV-BSs must dynamically adjust their positions, subject to the time-varying distribution of ad hoc ground users, to provide seamless wireless coverage and the maximum system capacity simultaneously [9]. An inappropriate UAV-BS coverage layout would result in a remarkable degradation in the system capacity.

### 1.1. Related Works

In the existing UAV-assisted wireless communication network, the UAV-BSs usually act as the backups of terrestrial BSs and adopt a circular coverage layout to provide wireless connectivity over certain areas when ground BSs are unavailable [9]. In addition, UAVs also play a vital role in robotic wireless sensor networks, where a UAV can gather sensed data from ground sensing nodes throughout its flying trajectory [10]. Based on such a circular coverage layout, the positions of the UAV-BSs can be optimized to achieve the best coverage [11,12,13], the highest system thoughput [14,15,16], or the maximum system energy efficiency [17,18,19].

The radio-coverage optimization for a UAV-BS has been quite intriguing to researchers recently [20,21]. In [20], a semi-progressive offloading deployment scheme was proposed so that the UAV’s down-tilt or altitude could be adjusted to achieve the maximum coverage and the minimum overlap. This scheme is able to reduce the overlapping interference while maintaining effective communication coverage. However, the proposed deployment scheme in [20] is highly dependent on terrestrial macro base stations (MBSs), and the UAV-BS can just be treated as a supplement for enhancing the communication coverage of MBSs. In [21], a placement scheme was proposed to maximize the communication coverage for UAV-BSs. The optimization problem was formulated to maximize the coverage of multiple UAV-BSs in a given area by adjusting their deployment positions while considering collision avoidance between the UAV-BSs. The optimization problem was solved by adopting the simulated annealing algorithm. It is noteworthy that the battery constraints of the UAV-BSs were not taken into account in [21]. On the other hand, the throughput was a common optimization objective in [22,23,24]. In [22], a communication model was proposed where UAVs are utilized to provide broadband coverage for blind zones in maritime communication networks. An optimization problem was formulated to maximize the minimum average throughput among all users by jointly optimizing user association, power allocation, and the UAV trajectory. To solve this problem efficiently, it was decomposed into three subproblems: user association optimization, power allocation optimization, and UAV trajectory optimization. A local optimal solution with low computational complexity was obtained using the successive convex approximation and block coordinate descent methods. The optimization technique in [22] considers the impact of the maritime environment and is based on an approximation method to handle the complexity of the two-ray channel model. In [23], a UAV was used by a mobile relay aviation base station to provide communication services in disaster areas. The system throughput was optimized by adjusting the height of the mobile relay. The optimization problem is a non-deterministic polynomial problem and is solved using an improved particle swarm optimization algorithm. However, the proposed approach in [23] ignores the horizontal mobility of the UAV, and in reality, the movement of a UAV can be more complex. A throughput maximization approach for improving communications in UAV-assisted networks by optimizing the UAV trajectory was proposed in [24]. This approach uses a graph neural network (GNN) to dynamically repurpose available UAVs to serve congested and overburdened areas of the network. In the scenario set-up in [24], the ground area was divided into several square cells, and the UAVs could serve in their designated cells. In [25], UAV-BSs could provide seamless communication service for ground users, and the total channel capacity was maximized by dynamically optimizing the UAV-BSs’ three-dimensional locations (coordinates). To accomplish this, the proposed Gibbs sampling distributed algorithm (GSDA) was employed in a distributed manner across all UAV-BSs, enabling each UAV to independently and asynchronously optimize its location.

In addition to the coverage and the throughput, there are many works aimed at other objectives for UAV-BS deployment optimization. The goal of [26] was to maximize the secrecy capacity of a UAV-enabled relaying system, which was achieved by jointly optimizing the UAV’s location, power allocation, and bandwidth allocation. This problem is formulated as a non-convex optimization problem and solved using their proposed successive convex approximation–alternative iterative optimization (SCA-AIO) algorithm. In [27], a deep Q network (DQN)-based learning model for optimizing the deployment of the UAV-BS was proposed. The model optimizes the trajectory of a UAV-BS by maximizing the mean opinion score (MOS) for the ground mobile users. In [28], the UAV trajectory was optimized to maximize the energy efficiency with a heuristic hexagon-based scheduling algorithm (HSA), which decomposed the network into hexagons. Table 1 summarizes the aforementioned related works.

However, when the UAV-BSs are deployed in a wide area for an ad hoc communication network, there inevitably exist blind spots within their service area once the circular coverage layout is adopted. In order to provide seamless coverage by a UAV-assisted ad hoc network, one can consider two types of polygonal cell shapes, namely squares and hexagons, to partition individual UAV-BS service areas for ground users. Both coverage configurations can indeed provide seamless service to ground users. Square cells can lead to an upper bound of the probability of coverage for the dense deployment of UAV-assisted terrestrial cellular networks [29]. However, due to the often dense deployment of UAV-BSs in a wireless network, the two aforementioned layouts would result in notable interference between a UAV-BS and the users served by its neighboring cells, which further decreases the total channel capacity. Thus, the coverage layout design to achieve the maximum total channel capacity of a UAV-assisted ad hoc network still remains intriguing.

### 1.2. Motivations, Contributions, and Limitations

With the ubiquitous need of multimedia and entertainment worldwide, there is an increasing demand for networks that are capable of supporting high-bandwidth applications, such as video live streaming and virtual reality (VR). In this regard, UAV-assisted networks have become increasingly popular, owing to their potential to meet such a demand. Since the total channel capacity is a crucial metric for evaluating the performance of a UAV-assisted network, it is essential to optimize the total channel capacity, particularly in remote or disaster-stricken areas, where terrestrial communication infrastructure is insufficient or impaired. Meanwhile, the square and hexagonal cell coverage layouts are commonly used for the optimization of UAV-assisted networks, as they are able to provide a seamless coverage area. In current works, the UAV position optimization algorithm is more complex when a hexagonal cell coverage layout is adopted, as there are more constraints to consider. However, one can save more cells at the boundaries of a service area when the hexagonal cell coverage layout is adopted over the square cell coverage layout. Consequently, it is of great importance to examine which of these two layouts exhibits superior performance in terms of total channel capacity. By determining which coverage layout is superior in terms of total channel capacity, one can further improve the optimization of UAV-assisted networks. In this work, the coverage layout of UAV-BSs is explored to maximize the total channel capacity of a UAV-assisted ad hoc network by use of an innovative channel capacity estimation method based on the path loss model. Our proposed new total channel capacity estimation method first evaluates the *mean distances* (MDs) between a UAV-BS and the ground users it serves as well as the MDs from these users to the UAV-BSs in neighboring cells. To reduce the necessary multiple integrals to double integrals, a novel polygon division strategy is devised in this work. Then, the total channel capacities of the UAV-assisted ad hoc network using two different coverage layouts are evaluated according to the two MDs stated above. In comparison with the conventional channel capacity calculation method, the computational complexity of our proposed new MD-based channel capacity estimation method is significantly lower. Our simulation results demonstrate that the square cell coverage layout of UAV-BSs can lead to a larger total channel capacity compared with the hexagonal cell coverage layout. This study could be very useful for the future deployment of UAV-assisted networks. It can guide people to select the coverage layout for optimal UAV-assisted network deployment. Note that our proposed new method for estimating the total channel capacity is based on the assumption that ground users can be densely and uniformly distributed within a service area. Hence, the mean distance from the ground users to the UAV-BSs can be adopted to derive a reasonable total channel capacity. The main contributions of this work are summarized as follows:

- A novel MD-based total channel capacity estimation method is designed for exploring the total channel capacity of various coverage layouts of a UAV-assisted ad hoc network.
- A new polygon division strategy is designed to reduce the computational complexity required for the calculation of MDs.
- We show that the square cell coverage layout can lead to a larger total channel capacity than the hexagonal cell coverage layout for UAV-assisted ad hoc networks.

The rest of this paper is organized as follows. Section 2 presents the basic system model and configuration of a UAV-aided wireless ad hoc network consisting of multiple UAV-BSs and ground users. Our proposed novel, computationally efficient channel capacity estimation approach is introduced in Section 3. The evaluation and comparison of the total channel capacities of a UAV-assisted ad hoc network using square and hexagonal cell coverage layouts are presented in Section 4. Finally, the conclusion will be drawn in Section 6.

*Nomenclature*: Scalars are denoted by italicized letters, such as *a*, vectors are denoted by letters with overhead arrow notions, such as $\vec{A}$, and sets are denoted by blackboard bold letters, such as A. The cardinality of a set A is denoted by |A|. $\vec{A}$^*T*^, which represents the transposes of a vector $\vec{A}$, and $∥\vec{A}∥$ denotes the Euclidean norm of a vector $\vec{A}$. The sets of all real numbers are denoted by R.

## 2. Problem Statement and System Model

For a UAV-assisted ad hoc wireless network, the ground coverage area can be seamlessly partitioned into polygons. This work investigates two common types of polygons used for such coverage layouts of UAV-BSs: squares and hexagons. These two layouts may lead to significant interference between a UAV-BS and the users served by its neighboring cells, which ultimately reduces the overall channel capacity. To further clarify the performance of the total channel capacity between the square cell coverage layout and the hexagonal cell coverage layout, the system model is described as follows.

**Figure 1:**
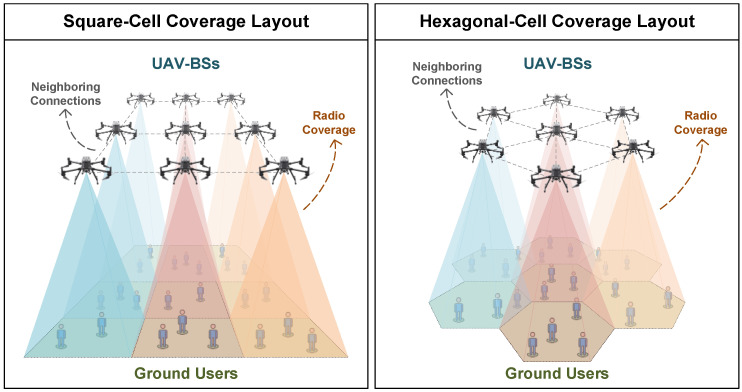
Two coverage layout scenarios of UAV-assisted ad hoc wireless networks. Each UAV-BS has a fixed serving area such that it can only serve the ground users within its radio coverage. Two typical coverage layouts are the square cell coverage-layout (**left**) and hexagonal cell coverage layout (**right**).

The corresponding scenarios of UAV-assisted ad hoc wireless networks and square and hexagonal cell coverage layouts are illustrated in Figure 1. In these scenarios, each UAV-BS has a fixed serving area such that it can only adjust its location within the boundary of the corresponding serving area. The ground users are randomly distributed within the coverage area. To evaluate the total channel capacities of these two network configurations, the network topology and the path-loss propagation model need to be established as follows.

### 2.1. Network Topology

**Figure 2:**
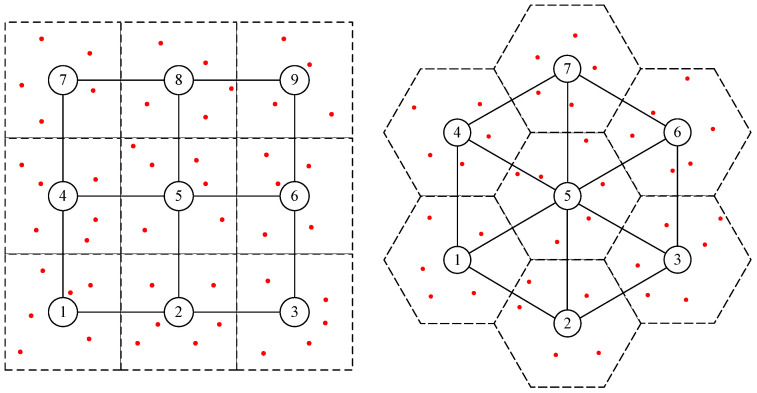
The configurations of the UAV-assisted ad hoc networks using square cell and hexagonal cell coverage layouts.

The ground coverage areas corresponding to the two different partitioned cells, as illustrated by Figure 1, are depicted in Figure 2. Suppose that J UAV-BSs (labeled by the circled numbers in Figure 2) are employed to serve I ground users (denoted by the red dots in Figure 2) in a UAV-assisted ad hoc wireless network, and the whole ground service area is seamlessly partitioned into (square or hexagonal) cells whose boundaries are highlighted by dashed polygons according to Figure 2.

The two-dimensional coordinates of the center of the jth UAV-BS’s ground coverage zone is denoted by ${\vec{\nu }}_{j}$$\overset{\mathrm{def}}{=}$${\left(x_{j}^{C},y_{j}^{C}\right)}^{T}$∈$R^{2\times 1}$, *j*=1, 2, *…*, J, where R represents a set of real numbers. Assume that each UAV-BS can only hover within its own coverage zone, and its three-dimensional coordinate vector can be obtained by its equipped global positioning system (GPS), which is denoted by ${\vec{L}}_{j}$$\overset{\mathrm{def}}{=}$${\left(x_{j}^{D},y_{j}^{D},h_{j}^{D}\right)}^{T}$∈$R^{3\times 1}$ for the jth UAV-BS $B_{j}$, *j*=1, 2, *…*, J. Then, a neighbor of $B_{j}$ is defined by a UAV-BS whose ground coverage zone is adjacent to that of $B_{j}$, and the neighborhood relationship among UAV-BSs in a UAV-assisted ad hoc wireless network remains unchanged. For $B_{j}$, *j* = 1, 2, where *…*, J, the index set of its neighbors, say $N_{j}$, can be written as

(1) $$N_{j}\overset{\mathrm{def}}{=}\left\{\bar{j}\;|\;B_{\bar{j}}\;\;\mathrm{is a neighbor of}\;B_{j},\bar{j}=1,2,\ldots ,J\right\},$$

where $\bar{j}$ is the index of a neighbor of $B_{j}$. The ith ground user $U_{i}$ with the two-dimensional coordinate vector ${\vec{\ell }}_{i}$$\overset{\mathrm{def}}{=}$${\left(x_{i}^{U},y_{i}^{U}\right)}^{T}\in R^{2\times 1}$, where *i*=1, 2, *…*, I is located within the coverage of the UAV-BS $B_{\kappa_{i}}$ such that

(2) $$\kappa_{i}\overset{\mathrm{def}}{=}\underset{j=1,2,\cdots ,J}{\mathrm{argmin}}\;∥{\vec{\ell }}_{i}-\vec{\nu_{j}}∥,$$

where ∥∥ denotes the Euclidean vector norm. Thus, the index set $U_{j}$ of all users covered by the UAV $B_{j}$ is given by

(3) $$U_{j}\overset{\mathrm{def}}{=}\left\{i\;|\;\kappa_{i}=j,\;i=1,2,\ldots ,I\right\}.$$

In practice, each UAV-BS can know all ground users’ locations (coordinates) to determine which users it needs to serve.

### 2.2. Path Loss Model

The *air-to-ground (ATG) path loss* for different environments is first characterized. The ATG path loss depends on a UAV’s altitude and the elevation angle between the UAV and its served user [30]. There involve two propagation classes, namely line-of-sight (LoS) and non-line-of-sight (NLoS) connections [30]. According to [30], the *mean ATG path loss* ${\mathrm{PL}}_{\zeta }$ (in dB) is given by

(4) $${\mathrm{PL}}_{\zeta }=\mathrm{FSPL}+\xi_{\zeta },$$

where FSPL denotes the *free space path loss* between a UAV and its served ground user, ζ∈{LoS,NLoS} specifies the type of connection, and $\xi_{\zeta }$ represents the *excessive path loss* due to an LoS or NLoS propagation channel between a UAV-BS (say $B_{j}$) and its served user (say $U_{i}$). Moreover, ${\mathrm{FSPL}}_{ij}$ is expressed by

(5) $${\mathrm{FSPL}}_{ij}=20\;\log_{10}\left(\frac{4\;\pi \;d_{ij}\;f}{c}\right),$$

where *f* specifies the carrier frequency of a transmitted signal, $d_{ij}$ denotes the distance between the transmitter (a UAV-BS $B_{j}$) and the receiver (a ground user $U_{i}$), and *c* represents the speed of light through air. According to [30], the probability of having an LoS connection between $B_{j}$ and $U_{i}$ can be expressed by

(6) $$P\left(\theta_{ij}|\mathrm{LoS}\right)=\frac{1}{1+a\;\exp\left[-b\left(\theta_{ij}-a\right)\right]},$$

where both *a* and *b* are the “*environment parameters*” such that

(7) $$\theta_{ij}\overset{\mathrm{def}}{=}\frac{180}{\pi }\times \tan^{-1}\left(\frac{\rho_{j}}{\sigma_{ij}}\right).$$

Note that $\rho_{j}$ specifies the altitude of a UAV-BS $B_{j}$ and $\sigma_{ij}$ denotes the projection of the distance between the UAV-BS $B_{j}$ and a user $U_{i}$ onto the ground plane. In addition, according to [30], the probability of having an NLoS connection between $B_{j}$ and $U_{i}$ can be expressed by

(8) $$P\left(\theta_{ij}|\mathrm{NLoS}\right)=1-P\left(\theta_{ij}|\mathrm{LoS}\right),$$

where $P\left(\theta_{ij}|\mathrm{LoS}\right)$ is given by Equation (6). Therefore, according to Equations (4)–(8), the expected path loss (measured in dB) between $U_{i}$ and $B_{j}$ is given by

(9) $$\Lambda_{ij}=\sum_{\zeta \in \{\mathrm{LoS},\mathrm{NLoS}\}}{\mathrm{PL}}_{\zeta }\times P\left(\theta_{ij}|\zeta \right).$$

### 2.3. Total Channel Capacity

The receiving power $\Psi_{i}$ of the ground user $U_{i}$ served by the UAV-BS $B_{j}$ where *j*=$\kappa_{i}$ and the total interference power $\Phi_{i}$ produced by all neighboring UAV-BSs $B_{\bar{j}}$, $\bar{j}$∈$N_{\kappa_{i}}$ under the uniform transmitting power $P_{T}$ (all UAV-BSs employ the same transmitting power $P_{T}$) can be expressed by

(10) $$\begin{array}{cc} \Psi_{i} & =P_{T}-\Lambda_{ij},\\ \Phi_{i} & =\sum_{\bar{j}\in N_{\kappa_{i}}}\delta \left(P_{T}-\Lambda_{i\bar{j}}\right), \end{array}$$

where $\Lambda_{ij}$ and $\Lambda_{i\bar{j}}$ denote the expected ATG path losses between a ground user $U_{i}$ and its serving UAV-BS $B_{j}$ as well as between $U_{i}$ and a neighboring UAV-BS $B_{\bar{j}}$, respectively, according to Equation (9). The interference and environment noise power $N_{i}$ can be defined by $N_{i}$$\overset{\mathrm{def}}{=}$$\vartheta [\Phi_{i}$+δ(N)], where N denotes the environmental noise power in dBm and δ() and ϑ() specify the power unit conversion functions between “dBm” and “Watt”, respectively, according to [31] such that

(11) $$\begin{array}{cc} D\;(\mathrm{in}\;\mathrm{Watt}) & =\delta \left(C\right)\overset{\mathrm{def}}{=}10^{\frac{C-30}{10}},\\ C\;(\mathrm{in}\;\mathrm{dBm}) & =\vartheta \left(D\right)\overset{\mathrm{def}}{=}30+10\;\log_{10}\left(D\right). \end{array}$$

Consequently, the received signal-to-interference-plus-noise ratio (SINR) of the ground user $U_{i}$ is given by

(12) $${\mathrm{SINR}}_{i}=\Psi_{i}-N_{i}.$$

According to [32,33,34,35], the *total channel capacity* $R_{j}$ for all ground users served by a UAV-BS $B_{j}$ can be calculated as follows:

(13) $$R_{j}=\sum_{i\in U_{j}}\log_{2}\left(1+{\mathrm{SINR}}_{i}\right),$$

where $U_{j}$ is defined by Equation (3).

## 3. Proposed Computationally Efficient Channel Capacity Estimation Scheme

In this section, we propose a computationally efficient channel capacity estimation scheme that can significantly reduce the computational complexity for evaluating the total channel capacities using two different coverage layouts. In the meantime, a polygon division strategy is designed for approximating the mean distances.

### 3.1. Mean Distance

The total channel capacity $R_{j}$ for all ground users served by $B_{j}$, as given by Equation (13), is related to the received SINRs of all such ground users. Consider Equations (4) and (5), where all of the distances between the ground users and their serving UAV-BSs as well as the distances between the ground users and their neighboring UAV-BSs must first be measured to obtain the received SINRs, which would lead to an enormous computational burden. In this work, the distribution of the ground users within the coverage zone served by a certain UAV-BS is formulated and then utilized to enumerate the means of the two aforementioned types of distances.

Assume that the ground user $U_{i}$ is evenly distributed within the coverage zone $\Omega_{\kappa_{i}}$ of their serving UAV-BS $B_{\kappa_{i}}$, which complies with a two-dimensional homogeneous point process. The probability density function $f_{X,Y}\left(x,y\right)$ of a ground user’s location can thus be formulated as follows:

(14) $$f_{X,Y}\left(x,y\right)\overset{\mathrm{def}}{=}\left\{\begin{array}{cc} \frac{1}{S_{\Omega }},\;\; & \mathrm{if}\;\left(x,y\right)\in \Omega_{\kappa_{i}},\\ 0,\;\; & \mathrm{if}\;\left(x,y\right)\notin \Omega_{\kappa_{i}}, \end{array}\right.$$

where $S_{\Omega }$ is the area of the coverage zone $\Omega_{\kappa_{i}}$ (Such an area is identical over all partitioned cells.) and (x,y) specifies a random user $U_{i}$’s location. Assume that the two-dimensional coordinate of the UAV-BS $B_{j}$ projected onto the ground plane is (m,n), and the projection distance between the user $U_{i}$ and the UAV-BS $B_{j}$ can be calculated as $\sigma_{ij}$=$\sqrt{{\left(x-m\right)}^{2}+{\left(y-n\right)}^{2}}$, which can be deemed a random variable. Thus, the conditional mean of $\sigma_{ij}$, subject to the UAV-BS’s location (m,n), can be expressed by

(15) $$E\left[\sigma_{ij}|\left(m,n\right)\right]=\underset{\Omega_{\kappa_{i}}}{\;\int \int \;}\sigma_{ij}\;f_{X,Y}\left(x,y\right)\;dx\;dy.$$

Since a UAV-BS can dynamically adjust its position within its coverage zone according to its served users’ movements, the projected two-dimensional ground position (m,n) of a UAV-BS $B_{j}$ also complies with a uniform distribution. Consequently, the probability of the UAV-BS $B_{j}$ within the zone $\Omega_{j}$ is given by $f_{M,N}\left(m,n\right)$$\overset{\mathrm{def}}{=}$$1/S_{\Omega }$, where $S_{\Omega }$ is also the area of the coverage zone $\Omega_{j}$. The ultimate mean of $\sigma_{ij}$ can thus be given by

(16) $$\begin{array}{ccc} E[\sigma_{ij}] & = & \underset{\Omega_{j}}{\;\int \int \;}E\left[\sigma_{ij}|\left(m,n\right)\right]\;f_{M,N}\left(m,n\right)\;dm\;dn\\ & = & \underset{\Omega_{j}}{\;\int \int \;}\underset{\Omega_{\kappa_{i}}}{\;\int \int \;}\sigma_{ij}\;f_{X,Y}\left(x,y\right)\;f_{M,N}\left(m,n\right)\;dx\;dy\;dm\;dn. \end{array}$$

The analytical solution to Equation (16) is too complex to obtain. Instead, we would like to approximate $E[\sigma_{ij}]$ here. Let us partition the coverage zone $\Omega_{j}$ into K equally spaced grid points with the coordinates $\left(m^{\left(k\right)},n^{\left(k\right)}\right)$, where *k* = 1, 2, *…*, K. Therefore, $\sigma_{ij}^{\left(k\right)}$$\overset{\mathrm{def}}{=}$$\sqrt{{\left[x-m^{\left(k\right)}\right]}^{2}+{\left[y-n^{\left(k\right)}\right]}^{2}}$. Consequently, we have

(17) $$E\left[\sigma_{ij}\right]\approx {\bar{\sigma }}_{j}^{K}\overset{\mathrm{def}}{=}\frac{1}{K}\;\sum_{k=1}^{K}\;\underset{\Omega_{\kappa_{i}}}{\;\int \int \;}\sigma_{ij}^{\left(k\right)}\;f_{X,Y}\left(x,y\right)\;dx\;dy.$$

**Figure 3:**
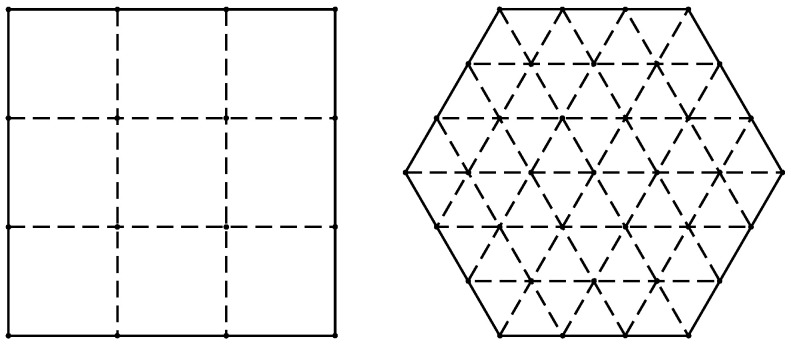
Illustration of the typical partitions of a square cell (**left**) and a hexagonal cell (**right**) for η = 3.

Note that $\lim_{K\to \infty }{\bar{\sigma }}_{j}^{K}$=$E[\sigma_{ij}]$. The typical partitions for a square cell and a hexagonal cell are illustrated by Figure 3. This polygon division strategy involves the construction of a grid system composed of the line segments that are parallel to each side of the polygon and the partitioning of a square or hexagonal cell into smaller equally sized squares or triangles. The coordinates of the vertices of the smaller squares and triangles, as shown in Figure 3, can be those of $\left(m^{\left(k\right)},n^{\left(k\right)}\right)$, as mentioned earlier. Let us denote the total number of partitioned segments of an external edge for a square or hexagonal zone by η (η = 3 for both the square and hexagonal zones in Figure 3). Hence, the total number of grid points is given by

(18) $$\begin{array}{c} K=\left\{\begin{array}{cc} {\left(\eta +1\right)}^{2},\;\; & \mathrm{for a square zone}\;\Omega_{j},\\ 3\;\eta^{2}+3\;\eta +1,\;\; & \mathrm{for a hexagonal zone}\;\Omega_{j}. \end{array}\right. \end{array}$$

**Figure 4:**
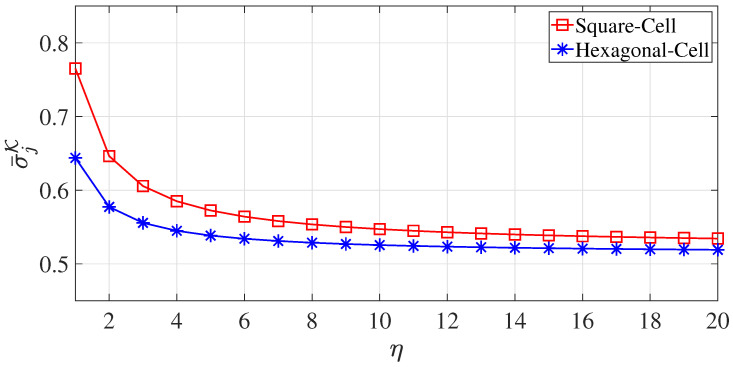
The convergence trends of ${\bar{\sigma }}_{j}^{K}$ with respect to η for both square and hexagonal cells (zones).

Heuristically speaking, when K≥20, ${\bar{\sigma }}_{j}^{K}$, given by Equation (17), converges to a constant value, as illustrated by Figure 4. Without loss of generality, we set each coverage zone (cell) to have a unit area (for both square and hexagonal zones) (i.e., the zone (cell) area $S_{\Omega }$ was one).

### 3.2. Total Channel Capacity Estimation

Once the mean distance from a ground user to its serving UAV-BS is determined (approximated) according to Equation (17), the total channel capacity can be estimated through it. The mean propagation distance from the users to their serving UAV-BS $B_{j}$ can be represented by ${\bar{d}}_{j}$$\overset{\mathrm{def}}{=}$$\sqrt{{\left({\bar{\sigma }}_{j}^{K}\right)}^{2}+{\left(\rho_{j}\right)}^{2}}$. Thus, according to Equation (5), the *mean free space path loss* from $B_{j}$ is given by

(19) $$\begin{array}{c} {\bar{\mathrm{FSPL}}}_{j}=20\;\log_{10}\left(\frac{4\;\pi \;{\bar{d}}_{j}\;f}{c}\right). \end{array}$$

Since the *mean ATG path loss* is given by Equation (4), according to Equation (9), the mean path loss from the UAV-BS $B_{j}$ to its served and neighboring ground users is expressed by

(20) $$\begin{array}{c} {\bar{\Lambda }}_{j}=\sum_{\zeta \in \{\mathrm{LoS},\mathrm{NLoS}\}}{\mathrm{PL}}_{\zeta }\times P\left({\bar{\theta }}_{j}|\zeta \right), \end{array}$$

where $P\left({\bar{\theta }}_{j}|\zeta \right)$ is equal to $P\left({\bar{\theta }}_{ij}|\zeta \right)$ by substituting $\theta_{ij}$ with ${\bar{\theta }}_{j}$ in Equations (6) (for ζ = “LoS”) and (8) (for ζ = “NLoS”) and ${\bar{\theta }}_{j}$ denotes the mean elevation angle of the ground users served by $B_{j}$ such that

(21) $$\begin{array}{c} {\bar{\theta }}_{j}=\frac{180}{\pi }\times \tan^{-1}\left(\frac{\rho_{j}}{{\bar{\sigma }}_{j}^{K}}\right). \end{array}$$

According to Equations (10) and (20), the mean received power $\bar{\Psi }$ of the ground users served by $B_{j}$ and the mean total interference power $\bar{\Phi }$ produced by all neighboring UAV-BSs $B_{\bar{j}}$ values are

(22) $$\begin{array}{c} \begin{array}{cc} \bar{\Psi } & =P_{T}-{\bar{\Lambda }}_{j},\\ \bar{\Phi } & =\left|N_{j}\right|\times \delta \left(P_{T}-{\bar{\Lambda }}_{\bar{j}}\right), \end{array} \end{array}$$

where $\left|N_{j}\right|$ denotes the total number of the neighboring UAV-BSs of $B_{j}$. The mean interference and environment noise power are thus given by $\bar{N}$$\overset{\mathrm{def}}{=}$$\vartheta \left[\bar{\Phi }+\delta \left(N\right)\right]$. The mean received SINR of a ground user served by $B_{j}$ can thus be expressed by

(23) $$\begin{array}{c} \bar{\mathrm{SINR}}=\bar{\Psi }-\bar{N}. \end{array}$$

The estimated *total channel capacity* $R_{j}$ for all ground users served by a UAV-BS $B_{j}$ can therefore be calculated as follows:

(24) $$\begin{array}{c} R_{j}\approx \left|U_{j}\right|\times \log_{2}\left(1+\bar{\mathrm{SINR}}\right), \end{array}$$

where $\left|U_{j}\right|$ denotes the total number of ground users served by $B_{j}$.

**Figure 5:**
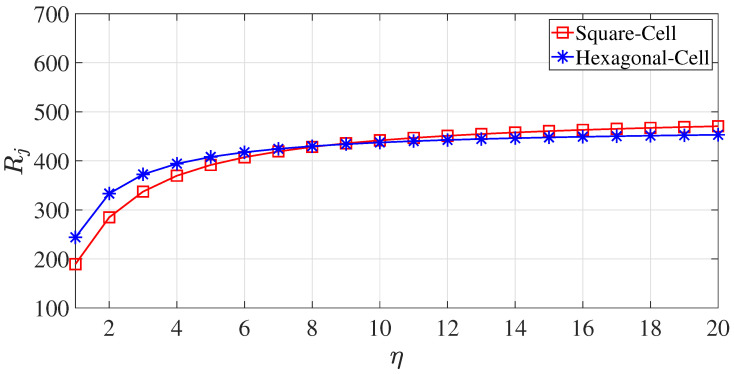
The convergence of $R_{j}$ with respect to η for both square and hexagonal cells (zones).

The total channel capacity was derived from the mean distance, and the convergence of the mean distance ${\bar{\sigma }}_{j}^{K}$ with respect to η was already verified in Section 3.1. Figure 5 illustrates the convergence of the total channel capacity $R_{j}$ with respect to η.

By incorporating the mean distances into the estimation of the total channel capacity, the computational complexity was significantly reduced. The numbers of various arithmetic operations involved in Equations (13) and (24) are listed in Table 2 for comparison.

Suppose that each UAV-BS serves a hundred ground users, and square and hexagonal cell coverage layouts are both considered. According to Table 2 and [36], when our proposed total channel capacity estimation method, given by Equation (24), is undertaken by an Intel Xeon processor running at 2.8 GHz, it takes 590 milliseconds (for square cells) and 824 milliseconds (for hexagonal cells) to calculate the total channel capacity using Equation (13), in comparison with 2.39 milliseconds (for both square and hexagonal cells) when using Equation (24) instead.

## 4. Simulations

In this section, the channel capacities of the UAV-assisted ad hoc network using the square and hexagonal cell coverage layouts are evaluated by our proposed new MD-based channel capacity estimation method in comparison with the conventional method. The simulation scenario was set up based on Figure 2, with one serving UAV-BS (labeled as “5”) and neighboring UAV-BSs (labeled as “2”, “4”, “6”, and “8” in a square cell coverage layout and labeled as “1”, “2”, “3”, “4”, “6”, and “7” in a hexagonal cell coverage layout). Here, we defaulted to the cell area being $S_{\Omega }\;=\;2\;\Theta^{2}$, making it identical for both the square and hexagonal cells such that the cell radii were Θ and $\Theta \sqrt{4/(3\sqrt{3})}$ for the square and hexagonal cells, respectively. Meanwhile, 100 users were randomly distributed over the center-most cell (labeled as “5”) in Figure 2. Every UAV-BS was located above its serving zone with a steady height of 100 m and moved only over the corresponding horizontal plane. Note that every UAV-BS could not get out of its zone boundary. Aside from that, each UAV-BS’s transmitting power was set to $P_{T}$ = 30 dBm. The minimum required received power of each ground user was $P_{\min}$ = −70 dBm. The carrier frequency of the transmitted signal was *f* = 3.5 GHz. The environment noise power was N = −100 dBm. Moreover, to determine the environment parameters *a* and *b* involved in Equation (6), we employed the numerical values of $\xi_{\mathrm{LoS}}$ and $\xi_{\mathrm{NLoS}}$, which are necessary for Equation (4), according to [30,37], for different environments. Here, we chose a suburban environment (*a* = 4.88, *b* = 0.43, $\xi_{\mathrm{LoS}}$ = 0.1, and $\xi_{\mathrm{NLoS}}$ = 21) for illustration.

**Figure 6:**
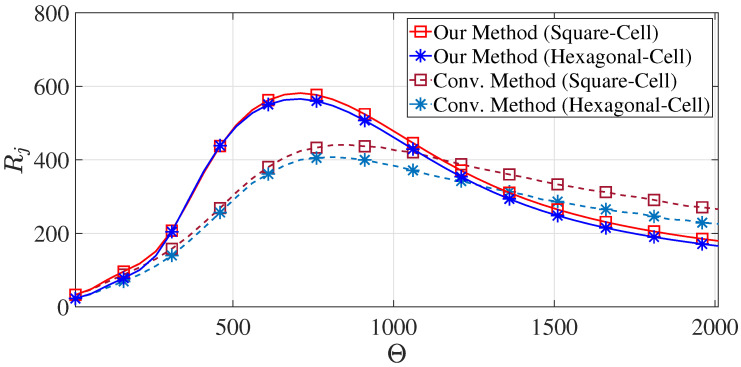
The total channel capacities with respect to Θ for both square and hexagonal cells with the identical cell area $2\;\Theta^{2}$.

In the conventional method, the ground users are considered to be uniformly distributed over the aforementioned center-most cell, and the distances from every user to its serving UAV-BS and its neighboring UAV-BSs are measured for computing the total channel capacity using Equation (13). On the other hand, our proposed new method only needs to calculate the two MDs for estimating the total channel capacity. In our simulation, 500 Monte Carlo trials were carried out under the aforementioned setting, and the average total channel capacities were computed with respect to the cell radii Θ ranging from 10 m to 2000 m for the square and hexagonal cell coverage layouts using the two methods stated above. The simulation was carried out using MATLAB R2021b, and the results are depicted in Figure 6.

According to Figure 6, it is conspicuous that the square cell coverage layout could always lead to a higher total channel capacity than the hexagonal cell coverage layout across various cell radii. Specifically, by using our proposed method, the total channel capacity of the square cell coverage layout was 7.67% larger than that of the hexagonal cell coverage layout. On the other hand, when using the conventional method, the total channel capacity of the square cell coverage layout was 14.94% larger than that of the hexagonal cell coverage layout. In a UAV-assisted ad hoc network, the ground user suffered more interference produced by its neighboring UAV-BSs when using the hexagonal cell coverage layout than the square-cell coverage layout. The results demonstrated by Figure 6 justify such a phenomenon. Meanwhile, according to Figure 6, our proposed new total channel capacity estimation method and the conventional method both led to very similar capacity values, where the UAV-assisted ad hoc networks using the square and hexagonal cell coverage layouts could both achieve the maximum total channel capacities when the cell radius was within 700–900 m.

## 5. Discussion

The focused study of this paper was to evaluate and compare the total channel capacities of two types of coverage layouts, namely square and hexagonal cell coverage layouts, using both our proposed novel, computationally efficient channel capacity estimation scheme and the conventional method. In addition to the numerical results of the achievable total channel capacity discussed in the previous section, Figure 6 indicates that there are two distinct stages: one is the increasing stage (when the cell radius is less than 700 m), and the other is the decreasing stage (when the cell radius is above 900 m). In the first increasing stage, the main factor for the total channel capacity to increase is the weakening of interference from neighboring UAV-BSs. When the cell radius reaches a certain value (around 700–900 m), the main factor for the total channel capacity to decrease becomes the increasing communication path loss.

In real-world scenarios, UAV-BSs would often encounter location precision errors, which may cause a UAV-BS to cross the cell boundary and move to a neighboring cell. To evaluate the impact of the positioning accuracy on the degradation in total channel capacity, a random position offset, denoted by τ, is introduced here to the locations of the UAV-BSs in the simulation presented in Section 4. Similarly, 500 Monte Carlo trials were conducted in the simulation, and the corresponding degradation percentages of the maximum achievable total channel capacity are listed in Table 3. The typical accuracy of the global positioning system (GPS) was within 4.9 meters, according to [38]. Here, we considered three different positioning accuracies of 5 m, 10 m, and 100 m in Table 3 to study the effect of the positioning accuracy. According to Table 3, when the positioning error (accuracy) increased, the degradation of the achievable total channel capacity grew. Nonetheless, such a positioning accuracy (ranging from 5 m to 100 m) imposed very little effect on the maximum achievable total channel capacity, as reflected by Table 3.

## 6. Conclusions

In this paper, the total channel capacity of a UAV-assisted ad hoc network for the suburban environment was evaluated, and the impact of the coverage layout on the total channel capacity was investigated. A new mean distance-based channel capacity estimation method was proposed to greatly reduce the computational complexity. Meanwhile, a new polygon division strategy was designed for the calculation of mean distances. According to our simulation results, the square cell coverage layout led to a higher total channel capacity than the hexagonal cell coverage layout for UAV-assisted ad hoc networks. Our proposed new method for estimating the total channel capacity is based on the assumption that ground users can be densely and uniformly distributed within the service area. In the future, further investigations can be conducted to explore the total channel capacity estimation scheme under diverse ground user distributions, as well as the actual total channel capacities resulting from a given UAV-BS optimization algorithm when subjected to the square cell coverage-layout and the hexagonal cell coverage-layout.

## Figures and Tables

**Table 1:** Summary of related works.

No.	Ref.	Method	Technique(s)	Advantage(s)	Limitation(s)
1	[20]	semi-progressive UAV deployment scheme	ring placement algorithm and position adjustment algorithm	reducing overlapping interference while maintaining effective communication coverage	highly dependent on terrestrial MBSs
2	[21]	simulated annealing-based coverage optimization algorithm	simulated annealing	maximizing the coverage of multiple UAV-BSs while avoiding collision	battery constraints were not considered
3	[22]	joint user association, power allocation, and UAV trajectory optimization algorithm	successive convex approximation and interior point techniques	maximizing the minimum average throughput by jointly optimizing the user association, power allocation, and UAV trajectory	various maritime environments were not considered
4	[23]	particle swarm optimization-based throughput optimization	particle swarm optimization algorithm	maximizing the system throughput by adjusting UAV’s height	horizontal mobility of the UAV was ignored
5	[24]	UAV repurposing-based approach for throughput maximization, delay, and packet loss minimization	graph neural networks	maximizing the throughput while the approach can accommodate any number of aerial nodes	battery constraints were not considered
6	[25]	Gibbs sampling distributed algorithm	Gibbs sampling and distributed optimization	maximizing the total channel capacity by dynamically optimizing the UAV’s location	battery constraints were not considered
7	[26]	successive convex approximation–alternative iterative optimization algorithm	successive convex approximation	maximizing the secrecy capacity by jointly optimizing UAV’s location, power allocation, and bandwidth allocation	energy consumption and throughput were not compromised
8	[27]	deep Q network-based learning model, enabling the optimal deployment of a UAV-BS	deep Q network	maximizing the mean opinion score for ground users by optimizing the UAV trajectory	training for mobile ground users was not considered
9	[28]	heuristic hexagon-based scheduling algorithm	greedy algorithm	maximizing the energy efficiency by optimizing UAV trajectory while decomposing the network into hexagons	real-time scheduling was not considered

**Table 2:** Comparison of numbers of arithmetic operations.

Arithmetic Operation	Equation (13)	Equation (24)
Sum	$\left(11+9\right|N_{j}\left|)\;\right|U_{j}|-1$	19
Product	$\left(15+14\right|N_{j}\left|)\;\right|U_{j}|$	31
Exponential	$\left(3+\right|N_{j}\left|)\;\right|U_{j}|$	4
Logarithm	$\left(2+2\right|N_{j}\left|)\;\right|U_{j}|$	4
Inverse trigonometric	$\left(1+\right|N_{j}\left|)\;\right|U_{j}|$	2

**Table 3:** Achievable total channel capacity degradation percentages subject to different positioning accuracies.

Method	Positioning Accuracy τ		
	5 m	10 m	100 m
Our Method	0.0043%	0.0399%	2.8398%
Conventional Method	0.0024%	0.0298%	2.6573%

## Data Availability

Not applicable.
